# Adjuvant-Associated Peripheral Blood mRNA Profiles and Kinetics Induced by the Adjuvanted Recombinant Protein Candidate Tuberculosis Vaccine M72/AS01 in Bacillus Calmette–Guérin-Vaccinated Adults

**DOI:** 10.3389/fimmu.2018.00564

**Published:** 2018-03-26

**Authors:** Robert A. van den Berg, Laurane De Mot, Geert Leroux-Roels, Viviane Bechtold, Frédéric Clement, Margherita Coccia, Erik Jongert, Thomas G. Evans, Paul Gillard, Robbert G. van der Most

**Affiliations:** ^1^GSK, Wavre, Belgium; ^2^Centre for Vaccinology (CEVAC), Ghent University Hospital, Ghent, Belgium; ^3^AERAS, Rockville, MD, United States

**Keywords:** tuberculosis, vaccine, adjuvant system, innate immunity, interferon, transcriptome

## Abstract

Systems biology has the potential to identify gene signatures associated with vaccine immunogenicity and protective efficacy. The main objective of this study was to identify optimal postvaccination time points for evaluating peripheral blood RNA expression profiles in relation to vaccine immunogenicity and potential efficacy in recipients of the candidate tuberculosis vaccine M72/AS01. In this phase II open-label study (NCT01669096; https://clinicaltrials.gov/), healthy Bacillus Calmette–Guérin-primed, HIV-negative adults were administered two doses (30 days apart) of M72/AS01. Twenty subjects completed the study and 18 subjects received two doses. Blood samples were collected pre-dose 1, pre-dose 2, and 1, 7, 10, 14, 17, and 30 days post-dose 2. RNA expression in whole blood (WB) and peripheral blood mononuclear cells (PBMCs) was quantified using microarray technology. Serum interferon-gamma responses and M72-specific CD4^+^ T cell responses to vaccination, and the observed safety profile were similar to previous trials. Two different approaches were utilized to analyze the RNA expression data. First, a kinetic analysis of RNA expression changes using blood transcription modules revealed early (1 day post-dose 2) activation of several pathways related to innate immune activation, both in WB and PBMC. Second, using a previously identified gene signature as a classifier, optimal postvaccination time points were identified. Since M72/AS01 efficacy remains to be established, a PBMC-derived gene signature associated with the protective efficacy of a similarly adjuvanted candidate malaria vaccine was used as a proxy for this purpose. This approach was based on the assumption that the AS01 adjuvant used in both studies could induce shared innate immune pathways. Subjects were classified as gene signature positive (GS^+^) or gene signature negative (GS^−^). Assignments of subjects to GS^+^ or GS^−^ groups were confirmed by significant differences in RNA expression of the gene signature genes in PBMCs at 14 days post-dose 2 relative to prevaccination and in WB samples at 7, 10, 14, and 17 days post-dose 2 relative to prevaccination. Hence, in comparison with a prevaccination, 7, 10, 14, and 17 days postvaccination appeared to be suitable time points for identifying potentially clinically relevant transcriptome responses to M72/AS01 in WB samples.

## Focus on the Patient

### What Is the Context?

Tuberculosis (TB) remains a serious infectious and contagious disease in many areas of the world. New vaccines are required because although the currently available TB vaccine, Bacillus Calmette–Guérin (BCG), works reasonably well in children, it does not work well in adults. What can help in the development of a new vaccine is knowing those immediate effects of vaccination that can predict how well it will work. RNA molecules represent a potent and now accessible source of information that can potentially capture these immediate effects because they reflect how vast arrays of the body’s genes are reacting. If predictions can be made with this information, then this can shape the design of future clinical trials and potential improvements to vaccine composition.

### What Is New?

This study analyzed RNA expression in healthy young adult recipients of the candidate TB vaccine M72/AS01. The study showed that the suitable time points for measuring these immediate effects of the M72/AS01 vaccine were at 7, 10, 14, and 17 days after vaccination. For this, a novel approach was followed in which a gene signature identified for another vaccine formulated with the same adjuvant was used as a proxy for the immune responses induced by the adjuvant. This RNA expression could be measured using the relatively small volumes associated with whole-blood (WB) samples rather than purified extracts of WB.

### What Is the Impact?

The information from this study will help streamline and strengthen the analyses of future clinical trials of the M72/AS01 vaccine.

## Introduction

Tuberculosis (TB), caused by *Mycobacterium tuberculosis* (Mtb), is a major source of morbidity and mortality in disease-endemic settings, especially in adolescents and adults (1). Primary human infection is often contained by the host immune response such that the infection becomes latent. However, in a minority of individuals, TB disease can occur, due either to reactivation of latent infection or to reinfection (2–5). The only available TB vaccine, BCG, is given mainly as primary vaccination at birth, and can protect children against severe forms of childhood TB including meningitis and disseminated TB. However, BCG does not fully protect against pulmonary TB, the most prevalent form of TB in adults (6, 7). Therefore, the candidate vaccine M72/AS01 is primarily being developed to protect against TB disease in adolescents and adults living in TB-endemic regions, who may or may not have latent Mtb infection (8–15). M72/AS01 contains the M72 antigen, a recombinant fusion protein derived from the Mtb proteins Mtb32A and Mtb39A, and the Adjuvant System AS01 (11). M72/AS01 has been shown to have a clinically acceptable safety profile in healthy subjects, and to induce humoral M72-specific antibodies and CD4^+^ T cells (8–15).

Although there are no known immune correlates of vaccine-mediated protection against TB in humans, CD4^+^ T-cell effectors, and the cytokines interferon-gamma (IFNG) and tumor necrosis factor (TNF)-alpha may play a role in protection (16, 17). In vaccine clinical trials, antigen-specific T-cell frequencies and antibody concentrations are typically used to interpret responses to vaccination. However, in clinical and preclinical studies in which BCG confers protection against Mtb from natural infection or experimental challenge, T-helper 1 (T_h1_) cell responses to BCG are at best poor predictors of protection (17–22). Therefore, alternative approaches are needed for the identification of TB vaccine correlates of protection (23). These alternative approaches also should recognize that in large field efficacy trials, logistics prevent frequent sampling and lengthy processing of high blood volumes for peripheral blood mononuclear cell (PBMC) preparation thus favoring immunological assessments to be made directly from small blood draws.

In this context, systems biology analyses have begun to provide additional insights into TB pathology (24, 25). Analyses of WB transcriptome data have identified gene signatures which were associated with active TB (26–29) and which are sensitive to successful treatment (26, 27, 30). Systems biology approaches on blood-derived data have also been used to evaluate clinical responses to several different vaccines, including the live-attenuated vaccines (rubella, smallpox, yellow fever), a live-recombinant vaccine (adenovirus/HIV-1), and non-live vaccines (inactivated-influenza vaccines, polysaccharide-based meningitis vaccines, and pneumococcal vaccine) (31–39). In those studies, gene signatures have been associated with a known adaptive immune profile, or a putative protective immune function. Some of those signatures include interferon-inducible genes and have been identified in WB or PBMCs within 7 days after vaccination (31, 35–37, 39). In two recent studies, gene signatures associated with protective efficacy have been identified for the candidate malaria vaccines RTS,S/AS01 and RTS,S/AS02 (40, 41). Those signatures also have potential relationships with IFNG signaling.

The objective of this study was to identify those postvaccination time points for WB sampling that could be relevant for evaluating M72/AS01 in future clinical studies, especially in efficacy studies. Given that M72/AS01-mediated protection has not been established yet, there is no associated gene signature that could inform the time point selection. Hence, it was decided to test a novel approach and use an alternative gene signature that was hypothesized to reflect AS01-induced immune activation as a proxy to measure the immunological diversity in transcriptome profiles as a function of time. In parallel, changes in RNA expression in PBMC and WB were analyzed using blood transcription modules (BTMs) (31), which provide an important tool for transcriptomic analysis. This BTM analysis provides a broad description of the responses induced after vaccination.

The specific gene signature that was used as a proxy was identified from PBMC RNA expression (transcriptome) data from recipients of the candidate malaria vaccines RTS,S/AS01 or RTS,S/AS02 in a vaccine efficacy study (41). In that study, vaccinated subjects underwent controlled challenge with malaria sporozoites, 14 days after the third and final dose. The results suggested that RNA expression of the specific gene signature (referred to as the RTS,S signature) between the 3rd and 14th day after the 3rd dose, rather than the day of or the day after the 3rd dose, contributed most to discriminating between protection and non-protection after challenge. Thus in the present study, the transcriptome data were used as an immunological readout, classifying subjects as gene signature positive (GS^+^) or –negative (GS^−^). Positive- or negative gene signature would thus correspond to an RNA expression profile formerly associated with protection or non-protection, respectively, against malaria sporozoite challenge after vaccination with RTS,S/AS01 or RTS,S/AS02.

Two assumptions were made to justify the use of the RTS,S gene signature in this M72/AS01 vaccination study. First, the RTS,S gene signature was driven by an interaction between innate and adaptive responses to vaccination, including the innate responses to the immunostimulants MPL and QS-21, which are constituents of AS01 and AS02 (41, 42). Hence, the RTS,S gene signature would be used as a proxy for different types of immune response to a vaccine with an adjuvant containing MPL and QS-21, M72/AS01. Second, the time window for the analysis of 7–17 days after vaccination and relative to baseline would best capture interactions between innate and adaptive immune responses to vaccination. Therefore, this time window would not necessarily coincide with a period when changes in gene expression were at their greatest magnitude, which perhaps would be expected 1 day after vaccination (40, 43). No assumption was made concerning the relationship between positive and negative RTS,S gene signatures and potential protection against TB because the pathology of TB is obviously different to that of malaria.

The impact of the different structural properties of the RTS,S antigen (virus-like particle) and M72 (soluble protein) could be an important determinant of the success of this approach. If the response induced by the different antigens has an important impact on the gene signature, then it will be unlikely to find evidence supporting the validity of using the gene signature for M72/AS01. If on the other hand, the gene signature is predominantly driven by the adjuvant components, then this study could potentially provide supportive evidence.

Therefore, the present study was designed to evaluate the kinetics of RNA expression relative to baseline up to 17 days after two doses of M72/AS01 using BTMs and to determine the most informative time point or time window between 7 and 17 days postvaccination for the collection of WB samples for RNA expression analyses using the RTS,S gene signature. Because the RTS,S gene signature was derived from PBMCs, the assessment of RNA expression in WB was compared with the assessment made in PBMCs.

## Materials and Methods

### Study Conduct and Design

This open-label study (NCT01669096) was conducted at the Centre for Vaccinology (CEVAC), Ghent University Hospital, Belgium, between August 2012 and May 2013. The protocol, its amendments, and other relevant study documentation were approved by the Ghent University Hospital’s Institutional Review Board, and the study was conducted in accordance with International Conference on Harmonisation Guideline for Good Clinical Practice, the Declaration of Helsinki, and all applicable regulatory requirements.

All screened individuals provided written informed consent. Eligible participants were male or female (non-pregnant) healthy adults aged 18–50 years at the time of the vaccination. They had received BCG vaccination, were seronegative for human immunodeficiency virus-1, and had no history of TB disease or Mtb infection based on a Quantiferon TB Gold test negative as surrogate. All eligible participants were stipulated to receive the candidate vaccine M72/AS01_E_ (referred to as M72/AS01 in the article; GSK, Rixensart, Belgium) by intramuscular injection at Days 0 and 30. The recombinant antigen M72 (10 μg/dose) was supplied as a lyophilized pellet and reconstituted with (0.5 ml/dose) AS01_E_. One dose of AS01_E_ contains 25 µg MPL (3-*O*-desacyl-4′-monophosphoryl lipid A), 25 µg QS-21 (*Quillaja saponaria* Molina, fraction 21; licensed by GSK from Antigenics LLC, a wholly owned subsidiary of Agenus Inc., a DE, USA corporation) and liposomes.

The exclusion criteria were standard for this type of clinical trial: the use of any investigational or non-registered product (drug or vaccine) other than the study vaccine, from 30 days preceding the first dose of study vaccine through to study completion; the chronic administration (defined as more than 14 days in total) of immunosuppressants or other immune-modifying drugs within 6 months preceding the first vaccine dose; the administration of immunoglobulins and/or any blood products within 3 months preceding the first dose of study vaccine through to study completion; the administration of long-acting immune-modifying drugs from 2 years preceding the first dose through to study completion; a history of any reaction or hypersensitivity likely to be exacerbated by any component of the vaccine; any confirmed or suspected immunosuppressive or immunodeficient condition; a history of medically confirmed autoimmune disease; and the concurrent participation in another clinical study.

During the study, elimination from the per-protocol (PP) cohort for immunogenicity was considered if the subject incurred a condition that had the capability of altering their immune response or had an alteration of their initial immune status.

### Study Endpoints

**Figure 1:**
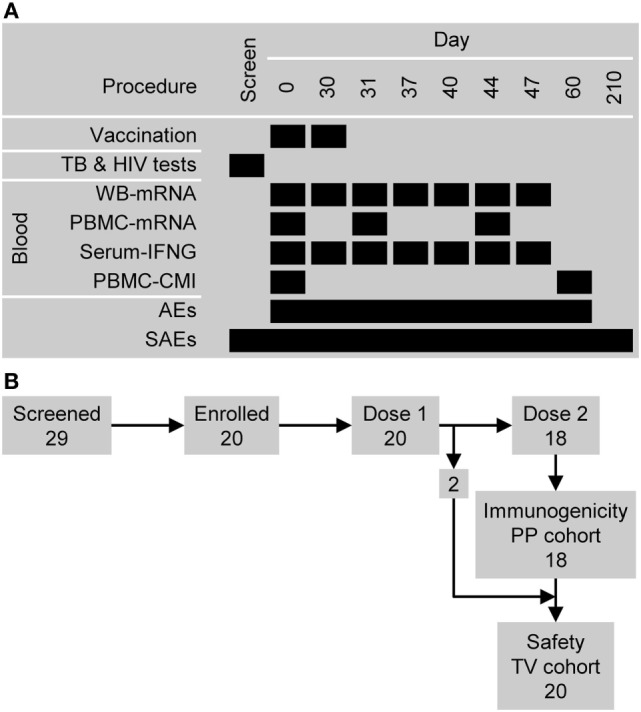
Study design overview. **(A)** The timing of vaccination and sampling procedures. Abbreviations: AEs, adverse events; CMI, cell-mediated immunity (i.e., antigen-specific CD4^+^ T cell frequencies); PBMC, peripheral blood mononuclear cell; SAEs, serious adverse events; and WB, whole blood. **(B)** Participant flow and the numbers entered into the immunogenicity per-protocol (PP) cohort and safety (total vaccinated, TV) cohort.

The immunogenicity research endpoints included the profiling of RNA expression using transcriptome microarrays with PBMC-derived samples on Days 0, 31, and 44; and with WB-derived samples on Days 0, 30, 31, 37, 40, 44, and 47 (Figure 1A). The profiling of RNA expression using RNA sequencing (for a comparison with using microarrays) has not been considered in this publication. Other immunogenicity endpoints included the evaluation of serum IFNG concentrations on Days 0, 30, 31, 37, 40, 44, and 47; and M72-specific T-cell (CD4^+^ and CD8^+^) frequencies per million T cells expressing at least two immune markers among CD40L, IFNG, IL2, TNF, IL13, or IL17 at Days 0 and 60. The safety endpoints included the occurrence of serious adverse events (SAEs) and potential immune-mediated diseases (pIMDs) during the entire study period; solicited injection site and general adverse events (AEs) within 7 days after each dose; and unsolicited AEs within 30 days after each dose.

### Safety

Adverse events and SAEs were identified in accordance with standard definitions. A subset of AEs that included autoimmune diseases and other inflammatory and/or neurological disorders of interest which may or may not have an autoimmune etiology were defined as pIMDs. Injection site AEs (pain, redness and swelling) and general AEs (fatigue, gastrointestinal symptoms, headache, malaise, myalgia and fever) were solicited daily from Days 0 to 6 after each dose. The intensity of injection site redness and swelling were assessed by measuring the diameters of the affected areas, and the intensity of fever was assessed by body (axillary) temperature. The intensities of other AEs, including unsolicited AEs were graded as follows: Grade 1, “easily tolerated” (“painful on touch” for injection site pain); Grade 2, “interferes with normal activity” (or “painful when limb is moved” for injection site pain); and Grade 3, “prevents normal activity” (or “considerable pain at rest” for injection site pain). An assessment of causality was made by the investigator for solicited systemic and unsolicited AEs, as well as for SAEs and pIMDs.

### Sample Preparation

At least 18 ml of blood was collected by venipuncture in lithium-heparin tubes (BD Biosciences, Erembodegem, Belgium), as described previously (44). Briefly, PBMCs were separated on Lymphoprep gradients, washed, counted by flow cytometry, frozen and further stored in liquid nitrogen until time of further evaluation. At least 10 ml of blood for WB gene expression analysis was collected in PAXgene tubes (PreAnalytiX, Hombrechtikon, Switzerland). Serum (for IFNG concentration) was prepared by centrifugation of at least 2 ml clotted blood sample (30 min to 1 h clotting time at room temperature) and stored at −70°C (±5°C).

### Serum IFNG Concentration Measurements

Serum IFNG was measured using BD Biosciences (Erembodegem, Belgium) cytometric bead array ES CBA and protocol. The assay’s lower limit of quantification (LLOQ) was set at 7.0 pg/ml: IFNG concentrations at or below this value were given an arbitrary value of 3.5 pg/ml.

### Cell-Mediated Immune Responses to Vaccination

Cryopreserved PBMCs were rapidly thawed and counted by flow cytometry using propidium iodide to identify dead cells. Cell recovery was calculated as the ratio of number of viable cells after thawing to number of cells before freezing. One million PBMCs were stimulated for 2 h by pools of overlapping peptides covering the entire M72 antigen sequence or medium only in the presence of anti-CD28/CD49d antibodies (used as costimulatory molecules), as described previously (44). Brefeldin A was added for a subsequent 18 h (overnight) incubation to promote intracellular accumulation of cytokines (44, 45). This antigen stimulation condition was optimal for expression of all the different cellular markers, both surface and intracellular, as described previously (44). Cells were stained using fluorochrome-conjugated antibodies before enumeration by flow cytometry. T cells were identified by positive expression of CD3. CD4^+^ T cells were typed as M72 specific when they expressed at least one of the following immune markers; CD40L, IFNG, IL2, TNF, IL13, or IL17. Acquisition was performed on a BD LSRII (Becton Dickinson) flow cytometer, and data were analyzed using the FlowJo software v.9.5.2 (Tree Star, Inc.). The assay’s LLOQ was set at a frequency of 1 M72-specific CD4^+^ T cell per million CD4^+^ T cells: frequencies below this value were given an arbitrary value of 1.

### Safety and Immunogenicity Data Analysis

Appropriate descriptive statistics were applied to demographic data, safety data, IFNG secretion data, and CD4^+^ T-cell frequency data using SAS version 9.2 (SAS Institute Inc., NC, USA) and StatXact-8.1 (Cytel, MA, USA) procedure on SAS.

### Profiling of RNA Expression

RNA was isolated from WB collected in PAXgene tubes (PreAnalytiX, Hombrechtikon Switzerland) or from isolated PBMCs using RLT buffer (Qiagen, Venlo, The Netherlands), and RNA was prepared using a standard Qiagen kit. RNA was amplified using the Ovation kit and protocol (NuGEN, CA, USA) and RNA expression levels were determined using the Human Genome-U133 Plus 2.0 arrays of 54120 probe sets derived from gene transcripts (Affymetrix, OH, USA).

### Microarray Data Preparation

The raw microarray data were normalized *via* GeneChip-Robust Multiarray Averaging (GC-RMA) (46), and outliers were excluded using the AffyPLM software package (open source; www.bioconductor.org, WA, USA) (47–49). For the PBMC samples, 4/54 microarrays were excluded, and for WB samples, 11/125 microarrays were excluded based on the relative deviation of the respective data sets using the NUSE method within the AffyPLM software package. The microarrays for WB samples from one subject were not processed because the Day 0 WB sample failed the quality control. After normalization, probe sets were filtered and retained, based on the interquartile range (>0.75) of RNA expression data (13.98% probe sets for PBMC samples and 12.63% probe sets for WB samples). The RNA expression data set can be accessed at the Gene Expression Omnibus^1^ under entry GSE102459.

### Gene-Set Enrichment Analysis of RNA Expression Data Based on Blood transcription Modules (BTMs)

Gene lists for the BTMs have been described previously (31). For this analysis, only those probe sets with annotated gene names were included. For each sample type (WB or PBMC) and for genes that were represented by more than one probe set, the data for the given gene (for all subjects) were selected from the probe set that presented the highest Pearson correlation with the scores of the first principal component from a principal component analysis of a matrix containing all data for the given gene (no scaling/no centering).

For each sample type (WB or PBMC) and for all genes, a linear mixed model (limma, R package) (50–52) was fitted to the RNA expression data, from which moderated *t* statistics were calculated for the comparison between each time point with baseline (Day 0, prevaccination) for the RNA expression of each gene. These *t* statistics were then used to calculate *p* values and false discovery rate (FDR) *p* values for these comparisons.

Genes were ranked (each sample type, each time point) based on absolute moderated *t* statistic values. The altered regulation of a BTM was identified by the enrichment (over representation at the top of the ranking) of genes corresponding to that BTMs using the CERNO test (using the R package *tmod*^2^; version 0.31) (53). The identification of the BTM was confirmed on condition that the RNA expression of the majority of genes within the BTM were significantly different from baseline (using the FDR *p* values; data not shown). Upregulation or downregulation of a BTM was determined by the relative prevalence of genes with RNA expression significantly higher or lower than baseline, respectively.

### Assignment of Subject Data Based on RNA Expression Related to Gene Signature

Individual subjects were assigned to positive and negative gene signature groups using RNA expression data. The procedure for group assignment is described in Section “Results.” The probe sets which defined the gene signature were classified into two clusters; Cluster A or Cluster B (see Table S1 in Supplementary Material). A one-sided Student’s *t*-test (α = 0.01) was used to identify differences between positive and negative gene signature groups at a particular time point in terms of overall gene (probe set) expression levels relative to baseline (Day 0). The procedure for group assignment was then repeated for bootstrapping and Monte–Carlo approaches based on the data sets generated by these procedures. A bootstrapping approach assessed the robustness of group assignment (54). Individual subject RNA expression data sets were drawn with replacement from the population of expression data sets to give a sample population equal in number and identically stratified in proportion with the original group assignments. A Monte–Carlo approach assessed the likelihood of arriving at the conclusion that overall expression levels were different (higher or lower) between positive and negative gene signature groups at a given time point using random data based on the observed distributions. First, the SD was calculated for the relative expression levels (to baseline) for all probe sets in a given cluster and all subjects regardless of group assignment. This SD was used to generate random data for all probe sets and all subjects by drawing random numbers from the following distribution, *N*(0,*s*_i_), where *s_i_* indicates the estimated SD of the genes in Cluster *i* (A or B). The procedure for group assignment was then repeated based on the random data. The resampling frequency or the Monte–Carlo frequency was the frequency in which a one-sided Student’s *t*-test identified a significant difference (α = 0.01) in overall gene expression levels relative to baseline between positive and negative gene signature groups in a given simulation for a total of 2,500 simulations.

## Results

### Study Conduct and Demography

Twenty-nine subjects were screened: five subjects did not satisfy the eligibility criteria and four subjects withdrew consent (Figure 1B). The 20 subjects who were enrolled had previously been vaccinated with BCG and were confirmed to be Quantiferon negative and HIV negative. All 20 subjects were vaccinated with M72/AS01 at least once and completed the study. Their ages ranged from 18 to 50 years with a median age of 28.5 years. Thirteen subjects were female and seven male. Ten subjects were of African heritage, nine were White Caucasian and of European heritage, and one was of East-Asian heritage. All 20 subjects were included in the safety set. Two subjects (1 male and 1 female) were not administered the second vaccine dose (due to Grade 2 myalgia and the administration of betamethasone, respectively), and hence, 18 subjects were included in the PP immunogenicity set.

### Safety

**Figure 2:**
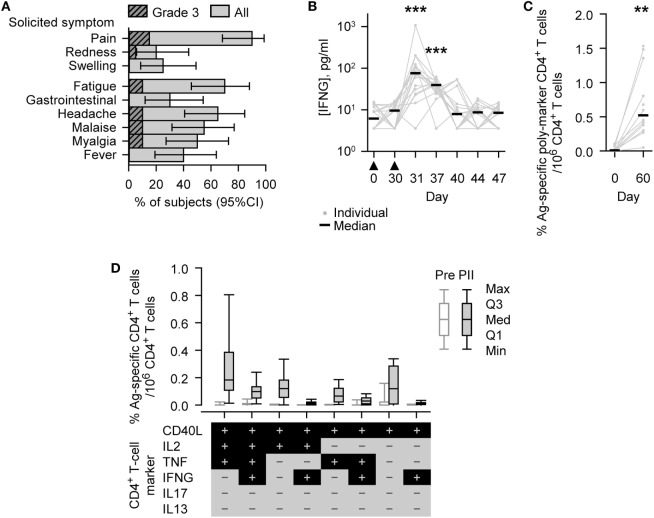
Safety and immunogenicity outcomes observed in vaccinated subjects. **(A)** Histograms describing the percentage of subjects (*N* = 20) reporting solicited adverse events (injection site symptoms and general symptoms of all grades or Grade 3 only) after either Dose 1 or Dose 2 during the 7-day (Days 0–6) postvaccination period. Grade 3 represents, for redness and swelling, a diameter >50 mm; for injection site pain, there is considerable pain at rest; for fever, an axillary temperature >39.5°C; and for other symptoms, normal activity is prevented. Gastrointestinal symptom is abbreviated to gastrointestinal. Error bars describe Fisher exact 95% confidence intervals (95% CIs). **(B)** Individual and median interferon-gamma (IFNG) concentrations of the evaluated immunogenicity cohort subjects (*N* = 18, Days 0 [Pre], 30 [PI], 37, 40, and 44; *N* = 17, Days 31 [1 day PII] and 47). The timing of vaccination is indicated below the x-axis by black triangles. **(C,D)** Antigen (M72)-specific CD4^+^ T-cell frequencies of the evaluated immunogenicity cohort subjects (*N* = 13, Day 0 [Pre]; *N* = 12, Day 60 [PII]). **(C)** Individual and median percentage frequencies of antigen-specific CD4^+^ T cells/million CD4^+^ T cells expressing two or more immune markers, or **(D)** Box and whisker plots describing the percentage frequencies of antigen-specific CD4^+^ T cells/million CD4^+^ T cells expressing defined combinations of immune markers (indicated below the *x*-axis) among CD40L, IL2, tumor necrosis factor (TNF), IFNG, IL13, and IL17 after short-term *in vitro* stimulation. The whiskers extend to the lowest (Min) and highest (Max) values; the box extends to the first quartile (Q1) and third quartiles (Q3) in which the median (Med) is marked by a horizontal line. Significance differences in panels **(B,C)** between postvaccination concentrations/frequencies and prevaccination concentrations/frequencies (Day 0) are indicated by asterisks (**p < 0.01; ***p < 0.001) and were determined by the Wilcoxon signed-rank test.

Injection site pain was the most frequent solicited AE and was reported by 18/20 subjects (90%). Redness and swelling were reported by 4/20 (20%) and 5/20 (25%) subjects (Figure 2A). Solicited general AEs of different types were reported by 6/20 (30%) to 14/20 (70%) subjects, with fatigue being the most frequently reported. Grade 3 pain was reported by 3/20 (15%) subjects, Grade 3 fatigue, headache, malaise, and myalgia were reported by 2/20 (10%) subjects in each case, and Grade 3 redness by 1/20 (5%) subjects. None of the Grade 3 symptoms lasted more than 4 days. Three solicited AEs were not related to treatment: Grade 1 myalgia reported by one subject and fever (37.6–38.0°C) reported by two subjects.

Twenty-three unsolicited AEs were reported by 12/20 (60%) subjects. Five unsolicited AEs related to vaccination were reported by 4/20 (20%) subjects, including symptoms classified under *General disorders and administration site conditions* (2 events), *Musculoskeletal and connective tissue disorders* (2 events), and *Nervous system disorders* (1 event). One of the *General disorders and administration site conditions* was graded 3.

Only one subject reported a serious adverse event (SAE), and this SAE was associated with alcohol abuse. No subjects reported a pIMD.

### Immunogenicity

Before vaccination, IFNG was detected (i.e., above the LLOQ) in the serum of 9/18 (50%) subjects (median concentration, 6.2 pg/ml; Figure 2B). At either 1 or 7 days after the second dose (Days 31 or 37), IFNG was detected in each of the 18 subjects; and at both of these time points, the concentrations of IFNG (medians; 76 or 39 pg/ml, respectively) were significantly higher than prevaccination (*p* < 0.001).

Before vaccination, M72-specific CD4^+^ T cells were detected in 9/13 (69%) subjects evaluated, with low median frequency of 97 marker-poly-positive CD4^+^ T cells (i.e., expressing at least two immune markers among CD40L, IL2, TNF, IFNG, IL13, and IL17) per million CD4^+^ T cells (Figure 2C). One month after the second dose (Day 60), the frequency of M72-specific marker-poly-positive CD4^+^ T cells (median, 5,200 per million CD4^+^ T cells) was significantly higher than prevaccination (*p* < 0.01). M72-specific CD4^+^ T cells were detected in all subjects evaluated, and the most prevalent CD4^+^ T cell phenotypes were all CD40L-positive and IL13/IL17 double-negative, and, in addition, generally either TNF/IL2 single or double positive, TNF/IL2/IFNG triple positive, or TNF/IL2/IFNG triple negative (Figure 2D; Figure S1 in Supplementary Material).

In the subjects evaluated, the median frequencies of CD8^+^ T cells per million CD8^+^ T cells expressing at least two immune markers were low before vaccination and 1 month after the second dose (95, Day 0 [*N* = 13] and 134, Day 60 [*N* = 12], respectively).

### Transcriptome Analysis by BTMs

**Figure 3:**
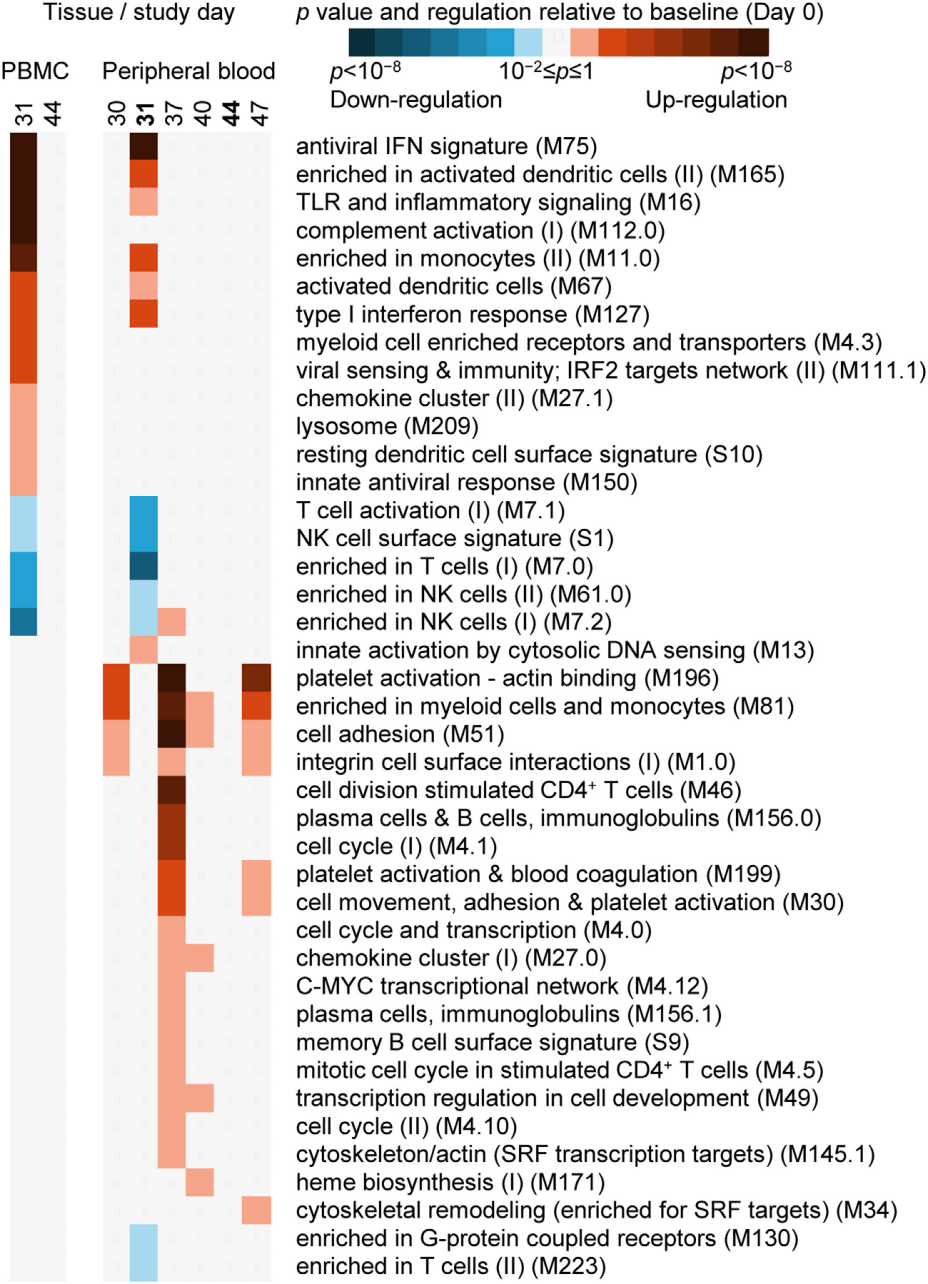
The evaluation of RNA expression changes relative to baseline (Day 0) at the level of blood transcription modules (BTMs, see Materials and Methods for definition) (31). A heatmap description of significant enrichment, with coloration indicating the directionality (upregulation or downregulation) of the majority of genes (coloration described in legend), in peripheral blood mononuclear cell (PBMC)-derived (left panel; Days 31 and 44), and whole-blood-derived (right panel; Days 30, 31, 37, 40, 44, and 47) RNA expression data from all study subjects evaluated. BTM titles and reference codes are described to the right of the heatmaps.

The recovery of RNA expression data sets was successful for most but not all samples from the 18 subjects in the immunogenicity cohort (see Materials and Methods). In PBMCs and WB, changes in RNA expression over time were identified in terms of the upregulation or downregulation relative to baseline (prevaccination) of BTMs using gene-set enrichment analysis (Figure 3). BTMs have been defined by associations with biological functions and/or tissue-specific expression patterns and provide an important tool for interpreting transcriptomic analysis by translating statistical outcomes, here changes compared to baseline, into functional enrichment (31). In the current analysis, 263 BTMs, comprising 9 to 347 genes, were investigated.

In the PBMC samples, 1 day after the second dose (Day 31), 13 BTMs were significantly upregulated, and 5 BTMs were significantly downregulated (Figure 3). Most upregulated BTMs were related to interferon signaling/antiviral sensing (four BTMs), dendritic cell and monocyte phenotypes (four BTMs), TLR/inflammatory signaling and chemokines (two BTMs). All five downregulated BTMs were related to T-cell or NK-cell phenotypes. At Day 44, no BTMs were significantly upregulated or downregulated.

In the WB samples 1 day after the second dose (Day 31), the phenotypes identified by BTMs were similar to but not as extensive as those identified in PBMCs (Figure 3), perhaps reflecting a greater level variation in RNA expression associated with a heterogeneous population of cells versus a purified (mononuclear) subpopulation of those cells. Seven BTMs were significantly upregulated, and seven BTMs were significantly downregulated. Six upregulated BTMs were also identified in the top seven significantly upregulated BTMs in the PBMC samples. Five of the seven downregulated BTMs were also identified as downregulated BTMs in the PBMC samples. Fourteen days after the second dose, and in common with the PBMC result, no BTMs were significantly upregulated or downregulated.

In WB and at Day 30 (the same day as the second dose) and at Days 37, 40, and 47, all of the 22 BTMs identified were up regulated, and nearly all (21/22) of these BTMs were distinct from those identified at Day 31 (Figure 3). Most (19/22) of these BTMs were up regulated at Day 37, and included phenotypes related to the cell cycle (6 BTMs), the cell cytoskeleton and adhesion (5 BTMs), and B cells and plasma cells (3 BTMs). At Days 30, 40, and 47 in comparison with Day 31, fewer BTMs were upregulated (4, 5, and 7 BTMs, respectively); and at Days 30 and 47, 3/4 and 4/7 BTMs were related to regulating the cell cytoskeleton and adhesion.

### Analysis of Suitable Time Points for Transcriptome Analysis

**Figure 4:**
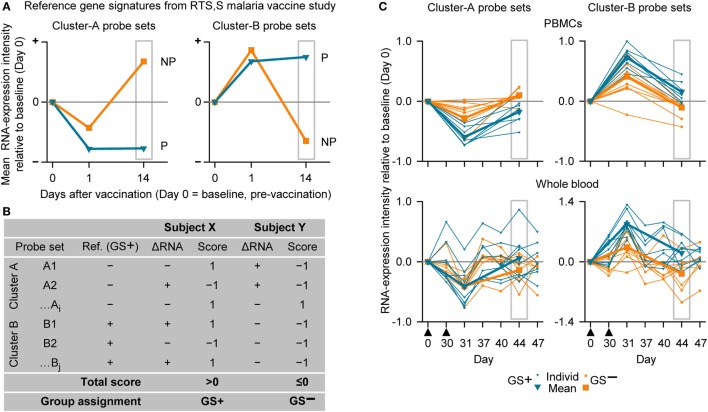
The assignment to gene signature-positive (GS^+^) and gene signature-negative (GS^−^) groups using RNA expression data from study subjects. **(A)** Reference gene signatures in RTS,S malaria vaccine efficacy trial (41) with respect to mean RNA expression intensities (levels) for the Cluster-A probe sets (left graph) and Cluster-B probe sets (right graph). Cluster A included 25 probe sets, and Cluster B included 40 probe sets (see Table S1 in Supplementary Material). The graphs show that the mean RNA expression levels differed, 14 days after vaccination, between vaccine recipients who were subsequently protected (P) or not-protected (NP) against malaria sporozoite challenge. **(B)** Voting system simulation for assignment of subjects to the GS^+^ and GS^−^ groups based on differences in RNA expression intensities with baseline (ΔRNA; higher [+] or lower [−]). Correspondence (both >0 or both <0) or no correspondence (one >0 and other <0) with a given GS^+^ reference ΔRNA of the probe set was scored +1 or −1, respectively. If the overall score for all probe sets from both clusters was above 0 then the subject was assigned to the GS^+^ group, and if the overall score was 0 or below then the subject was assigned to the GS^−^ group. **(C)** Individual and mean overall RNA expression intensities relative to baseline (Day 0) for Cluster-A probe sets (left graphs) and Cluster-B probe sets (right graphs) for the evaluated peripheral blood mononuclear cell (PBMC) samples (upper graphs) and whole-blood samples (lower graphs) from study subjects assigned to either the GS^+^ group or the GS^−^ group. Group assignment was based on PBMC RNA expression data at Day 44 (highlighted by gray rectangular outlines). Note that means are only shown for Days 0, 31, and 44. The timing of vaccination is indicated below the *x*-axes of the lower graphs by black triangles.

For our next analysis, we focused on identification of suitable time points. The identification of a time window for transcriptomic analysis using WB was based on the premise that the data generated should be able to distinguish between two different response patterns, i.e., GS^+^ or GS^−^, after M72/AS01 vaccination. The reference gene signature was based on 65 microarray probe sets representing at least 62 genes (see Table S1 in Supplementary Material). This gene signature was selected because in a clinical study of healthy adult recipients of the malaria vaccine (55), the kinetics of RNA expression in PBMCs detected by the probe sets could be used in mathematical models to distinguish the outcomes to subsequent malaria sporozoite challenge (i.e., GS^+^ versus GS^−^) (41). The 65 probe sets had been assigned to one of the two clusters (Clusters A and B) related to apparent reciprocal differences in RNA expression kinetics relative to baseline, such that at 14 days post-final dose, the change in RNA expression for each of the Cluster-A probe sets was negative in the GS^+^ group, but positive in the GS^−^ group, whereas the change in RNA expression for each of the Cluster-B probe sets was positive in the GS^+^ group, but negative in the GS^−^ group (Figure 4A). Therefore, the change in RNA expression at the 14-day post-last dose time point relative to baseline (either positive or negative) for each of these probe sets in the GS^+^ group were used as the references for the present study.

Thus, in the present study, a subject was assigned to GS^+^ or GS^−^ groups based on comparisons between their RNA expression data post-dose 2 and the reference RNA expression data of the GS^+^ group (Figures 4A,B). First, a score of 1 or −1 was allocated if the change in RNA expression relative to baseline (either positive or negative) for a given probe set was the same or different, respectively, as that for the reference in the GS^+^ group. Second, the overall score was determined from the sum of the scores for the entire list of probe sets (Cluster A and Cluster B). Hence, the subject was assigned to the GS^+^ group if the overall score was above 0; and assigned to the GS^−^ group if the overall score was 0 or below. Third, group assignment was then evaluated based on mean RNA expression levels relative to baseline for all probe sets within each of the clusters to quantify the robustness of the assignment.

In the preliminary analysis, the RNA expression data were evaluated from PBMCs at 14 days after the second dose (Day 44). About one half of the subjects evaluated (8/17) were assigned to the GS^+^ group. For both Cluster-A and Cluster-B probe sets, the assignments to the GS^+^ and GS^−^ groups presented significant differences between the groups in overall mean RNA expression levels at Day 44 (Figure 4C; Table 1). The assignments were supported by high frequencies of identifying differences through bootstrapping (95.3 and 100% for Cluster-A and Cluster-B probe sets, respectively). The Monte–Carlo simulations suggested that the frequency of identifying differences through randomly generating probe set expression levels and repeating group allocation would be 2.32 and 2.44% for Cluster-A and Cluster-B probe sets, respectively. Therefore, M72/AS01 vaccine recipients could be assigned to different vaccine-response groups based on Day 44 PBMC-derived RNA expression data using the selected Cluster-A and Cluster-B probe sets.

**Table 1:** Analysis of RNA expression differences between subjects assigned to the gene signature-positive group and subjects assigned to the gene signature-negative group.

Sample	Day	Cluster A probe sets			Cluster B probe sets		
		*p*-Value^a^	Bootstrap. freq. (%)	MC freq. (%)	*p*-Value	Bootstrap. freq. (%)	MC freq. (%)
PBMC	44	1.80×10^−5^	95.3	2.32	1.51×10^−18^	100	2.44
WB	37	2.73×10^−2^	42.8	1.92	6.67×10^−16^	99.3	1.80
WB	40	0.249	21.0	2.56	6.09×10^−44^	100	1.96
WB	44	0.968	1.28	2.04	5.23×10^−36^	100	1.88
WB	47	1.50×10^−4^	77.8	2.28	1.20×10^−8^	84.4	2.24

*Group assignment and RNA expression analysis was performed on the same RNA expression data set (by PBMC or WB and by time point)*.

*^a^One-sided Student’s t-test used to determine p-value: p < 0.01 was considered significant*.

*PBMC, peripheral blood mononuclear cell; freq., frequency; MC, Monte–Carlo; Bootstrap., Bootstrapping (resampling with replacement); WB, whole blood*.

When group assignment based on PBMC data at 14 days after the second dose (Day 44) was evaluated with respect to the RNA expression levels determined in WB samples at Day 44, the distinction between the GS^+^ and GS^−^ groups was apparent but only using the Cluster-B probe sets (Figure 4C).

**Figure 5:**
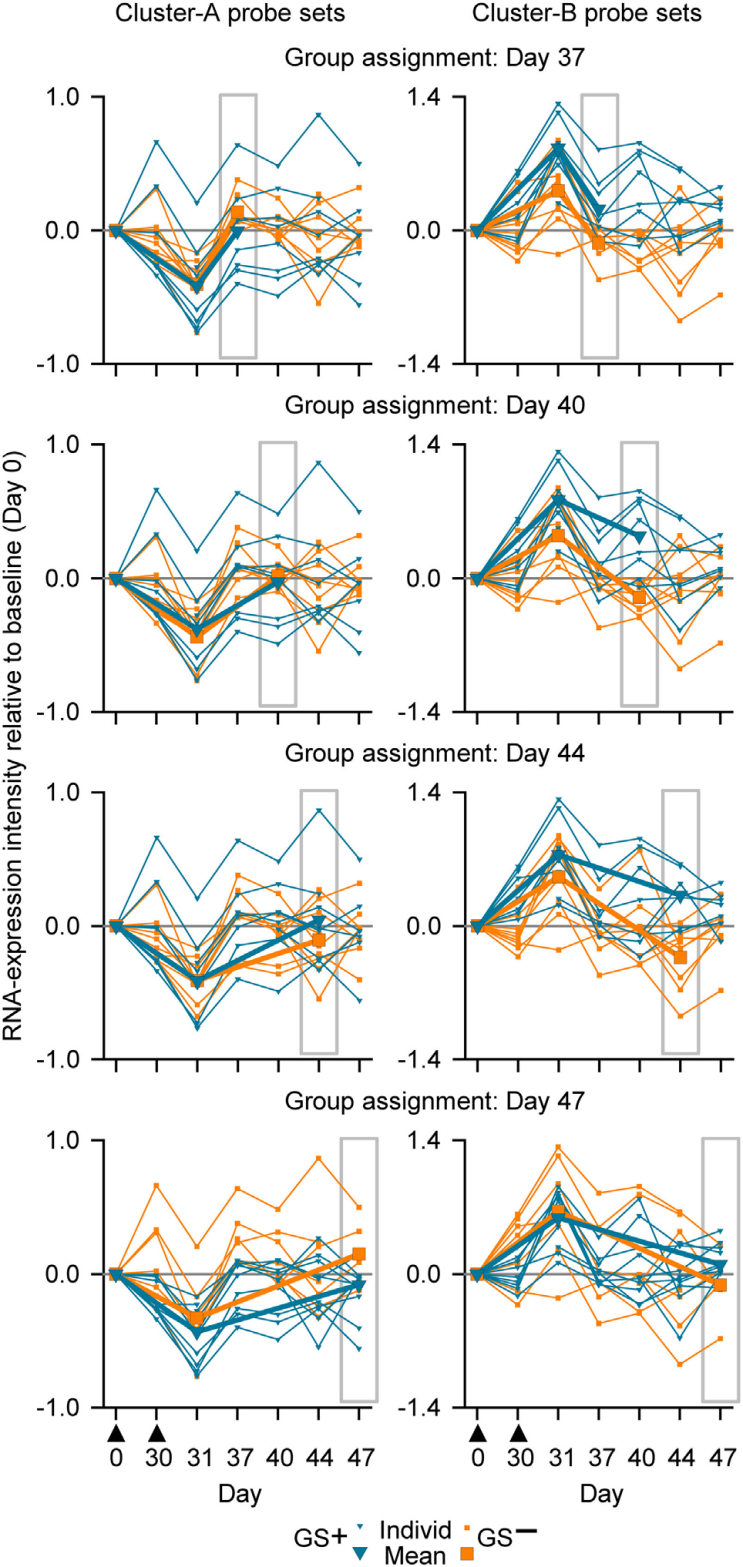
Individual and mean overall RNA expression intensities relative to baseline (Day 0) for Cluster-A probe sets (left graphs) and Cluster-B probe sets (right graphs) for the evaluated whole-blood (WB) samples from study subjects assigned to either the gene signature-positive (GS^+^) group or the gene signature-negative (GS^−^) group. Group assignment was based on WB RNA expression data at Days 37, 40, 44, and 47 (highlighted by gray rectangular outlines). Note that means are only shown for Days 0, 31, and the day from which group assignment was determined. The timing of vaccination is indicated below the *x*-axes of the lower graphs by black triangles.

In the main analysis, RNA expression data from WB were used to determine assignment to the GS^+^ and GS^−^ groups over a range of time points after the second dose (Days 37, 40, 44, and 47). At each of the time points, about a half of the subjects evaluated (8/17 or 10/17 [Day 47]) were assigned to the GS^+^ group. However, only three subjects were consistently assigned to the GS^+^ group and three subjects were consistently assigned to the GS^−^ group. For Cluster-B probe sets, the assignments to the GS^+^ and GS^−^ groups presented significant differences in overall mean RNA expression levels at all time points, and with high bootstrapping frequencies (84.4% [Day 47] to 100% [Days 40 and 44]; Figure 5; Table 1). By contrast, for Cluster-A probe sets, the GS^+^ and GS^−^ groups only presented a significant difference in overall mean RNA expression levels at Day 47 (*p* = 1.50×10^−4^), with a bootstrapping frequency at 77.8%. Therefore, in WB and PBMCs at all time points evaluated, Cluster-B probe sets consistently confirmed differences in RNA expression between the GS^+^ and GS^−^ groups. However, WB differed from PBMCs in that Cluster-A probe sets did not confirm differences in RNA expression between the GS^+^ and GS^−^ groups at Day 44.

In summary, our results suggested that sampling WB from any of the postvaccination time points evaluated (7, 10, 14, and 17 days post dose 2), in combination with a prevaccination time point, could yield differential transcriptomic profiles and could have the potential to inform on relevant biological differences in the responses to M72/AS01. However, a superiority of one sampling time point over the others was not obvious. Although for the 17-day time point in contrast to the other time points, the assignments to GS^+^ and GS^−^ groups were confirmed by both Cluster-A and Cluster-B probe sets, the three statistical measures for group assignments using the Cluster-B probe (*p* value, bootstrapping frequency and Monte–Carlo frequency) were relatively inferior to those for the other time points.

### Group Assignment and Immunogenicity

**Figure 6:**
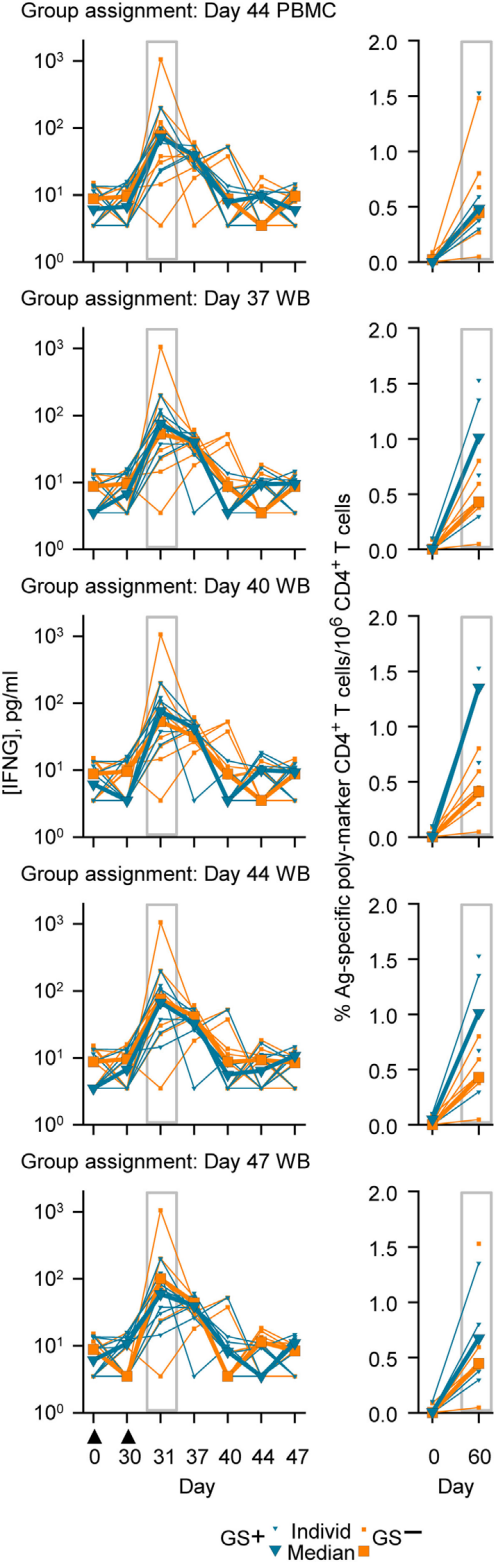
Individual and median serum interferon-gamma (IFNG) concentrations (left graphs) and individual and median frequencies of antigen (M72)-specific CD4^+^ T-cells/million CD4^+^ T cells (right graphs), in accordance with group assignment based on RNA expression data from peripheral blood mononuclear cell (PBMC) at Day 44 or whole blood (WB) at Days 37, 40, 44, and 47. The serum IFNG concentrations at Day 31 and the antigen (M72)-specific CD4^+^ T-cell frequencies at Day 60 are highlighted by gray rectangular outlines. The timing of vaccination is indicated below the *x*-axis of the lowest IFNG graph by black triangles.

The genes represented in the Cluster-A and Cluster-B probe sets included those genes associated with the IFNG pathway and potentially with lymphocyte function (41). Therefore, in this study a *post hoc* assessment of IFNG concentrations and antigen-specific poly-positive CD4^+^ T-cell frequencies was made based on the group assignments using PBMC or WB RNA expression data (Figure 6). Median concentrations of IFNG at Day 31 ranged between 59 and 76 pg/ml in the GS^+^ group and ranged between 53 and 101 pg/ml in GS^−^ group for the five different group assignment regimes. Median antigen-specific CD4^+^ T cell frequencies at Day 60 ranged between 4,852 and 13,539 per million CD4^+^ T cells in the GS^+^ group and between 4,107 and 4,471 per million CD4^+^ T cells in the GS^−^ group for the five different group assignments regimes. However, individual subject IFNG concentrations and CD4^+^ T-cell frequencies were highly variable prevaccination and postvaccination and the groups’ sizes were small, thus preventing any meaningful statistical comparison.

## Discussion

Peripheral blood-derived transcriptome data from vaccine clinical trials have the potential to provide predictive information about immunogenicity and protection in individual vaccine recipients and insight into the mode of action of a vaccine (31, 40, 41, 56). This study represents a preliminary step in the incorporation of transcriptome data analysis into subsequent M72/AS01 clinical trials. Moreover, as with previous studies in healthy adult subjects, M72/AS01 had an acceptable safety profile (8, 10, 11), and no safety concerns were identified.

To analyze the RNA expression data, two different approaches were utilized. First, a kinetic analysis of RNA expression changes using BTMs revealed early (1 day post-dose 2) activation of several pathways related to innate immune activation, both in WB and PBMC. Second, a novel approach was followed to identify optimal postvaccination time points using a previously identified gene signature that was hypothesized to reflect AS01-induced immune activation as a classifier. Because gene signatures associated with potential M72/AS01-mediated protection have yet to be determined, a gene signature associated with RTS,S vaccine-mediated protection against malaria (41) was used as a proxy to measure the immunological diversity in transcriptome profiles as a function of time. Although the use of a gene signature from a different vaccine as a reference was a limitation of the study, the gene signature’s relevance was based on it potentially capturing differing responses to the two immunostimulants, MPL and QS-21, that are used in the adjuvant systems for the candidate malaria vaccines (RTS,S/AS01 and RTS/AS02) and in M72/AS01. Because of the differences between malaria and TB disease, it was considered as overly speculative to explain the relevance of the proportion of vaccine recipients assigned to one or the other of the groups, or to explain the association between group assignment and the potential for protective responses against TB.

The conclusions of the study were limited by the study’s small size and that the cohort included only healthy BCG-vaccinated Quantiferon-negative adults resident in Belgium. Nevertheless, the RNA expression data analysis allowed two conclusions. First, the results of BTM enrichment analysis revealed that the early response to vaccination is characterized by activation of several pathway linked to innate immune activation. This transcriptomic response was transient and was succeeded by activation of BTMs related to adaptive immune responses. Second, this study demonstrated that RNA expression of a set of genes potentially relevant to the mode of action of the vaccine adjuvant could be used to discriminate different responses to the M72/AS01 vaccine using WB samples. These samples could be taken at 7, 10, 14, and 17 days postvaccination. Hence, transcriptome analysis of WB RNA at the same sampling time points may be relevant to evaluate immunogenicity, safety, and possibly protection, in the future M72/AS01 trials. The dynamic regulation of specific BTMs in the current study was similar to what has been observed in PBMC transcriptomes of recipients of the RTS,S/AS01 vaccine (40). These results support the argument that there are shared pathways induced by the immunostimulants in the AS01 and AS02 adjuvant systems and a common mode of action. Indeed, in both studies and 1 day after the second dose, pathways related to interferon signaling/antiviral sensing and dendritic cell and monocyte phenotypes were upregulated and phenotypes related to T cells and NK cells were downregulated. With the WB samples, the phenotypes identified by BTMs were similar but not as extensive as those identified in PBMC samples at the comparable time point of 1 day after the second dose. At 6–7 days after the second dose, phenotypes related to the cell cycle, and to CD4 T cells, B cells, and plasma cells were upregulated. These phenotypes also suggested a transition from innate to adaptive responses to vaccination. In AS01 mode of action studies in mice, AS01 enhances adaptive immune responses to the vaccine antigen by triggering innate immune activity at the injection site and draining lymph node including the transient release of cytokines, the transient release of IFNG (which can also be detected in peripheral blood), the recruitment and activation of monocytes and dendritic cells, and more effective antigen presentation by dendritic cells (57, 58). Elsewhere, it has been suggested that antigen-specific lymphocytes can be mobilized into the circulation by 7 days (59). The RTS,S gene signature analysis revealed that subjects can be classified in GS^+^ and GS^−^ groups. Hence, there are distinct different responses to M72/AS01 (marked by positive or negative gene signatures) that may reflect, in part, differences in innate immune activity triggered by AS01 and subsequent differences in adaptive immunity.

At the other comparable time point of 14 days after the second dose, assignments to GS^+^ and GS^−^ groups were confirmed only by Cluster-B probe sets in WB, but by both Cluster-A and Cluster-B probe sets in PBMCs. These differences may reflect that a greater level of variation in RNA expression associated with a more heterogeneous population of WB cells versus a purified (mononuclear) subpopulation of those cells with presumably, a lower prevalence of Cluster-A confounding cell types. These differences may also reflect that the reference gene signature, in being derived from PBMCs, was better suited to identifying significant difference in PBMCs and not WB from the M72/AS01 recipients. Hence, in WB samples, the consistency of subjects being assigned to the same GS group (GS^+^ or GS^−^) at the 4 time points may have been compromised by using Cluster-A probe set data. Nevertheless, the study suggested that potentially relevant transcriptome information can be retrieved from WB samples. And this is supported by other studies where potentially relevant biological differences have been identified with systems biology analyses of WB samples in TB disease (26–30) and in the evaluations of other vaccines (35, 37, 39).

No consistent association was observed between the preferential assignment to the GS^+^ group and higher IFNG concentrations or higher M72-specific immune marker poly-positive CD4^+^ T cell frequencies postvaccination. Overall, the increases after vaccination in serum IFNG concentrations, and in the frequencies of M72-specific CD4^+^ T cells with an immune marker poly-positive and T_h_1-bias phenotype were in agreement with earlier studies of the M72 vaccine in adults (8, 10, 11). The detection of M72-specific CD4^+^ T cells prevaccination presumably reflected the immune memory of BCG vaccination or possible cross-reactive non-Mtb mycobacteria exposure (11); and an immune memory may have contributed to the continued elevation of serum IFNG concentration 7 days after the second dose (41, 60).

The suitability of the sampling time points 7, 10, 14, and 17 days postvaccination, suggested by GS^+^/GS^−^ assignment had little correspondence with the numbers and types of BTMs identified at those time points. The potentially and relatively superior 17-day postvaccination time point by GS^+^/GS^−^ assignment was associated with fewer BTMs than the 7-day postvaccination time point; and no BTMs were identified at the 14-day postvaccination time point. Two explanations can be envisaged. First, the absence of any clear relationships between BTM and GS analyses may be linked to the fact that none of the 25 Cluster A genes and only 4/37 Cluster B genes were represented in the 41 identified BTMs (i.e., *IRF7, NCF1C, MYD88*, and *PML*). Second, differences in gene expression that are related to differences in immunogenicity or protection status may not necessarily coincide with those time points in which gene expression levels relative to baseline (as measured by BTM analysis) is maximally increased or decreased. This has been observed in recipients of the RTS,S vaccine (40), where NK-cell-related BTMs were negatively correlated with protection and immunogenicity on the day of the third RTS,S/AS01 dose, even though a significant downregulation was not detected on the same day (but was detected the day after).

In summary, M72/AS01 had an acceptable safety profile and was immunogenic in the small study population of healthy adults. All four time points evaluated (7, 10, 14, or 17 days postvaccination) in comparison with a prevaccination time point, appeared suitable for identifying potentially clinically relevant transcriptome responses to M72/AS01 in WB samples. Hence on condition that an appropriate reference gene signature can be identified, the approach taken in this study to use a gene signature associated with shared immune pathways, possibly reflecting a common mode of action, as a proxy for the induced immune response should also be suitable for other vaccine clinical studies that address the same type of objective.

## Ethics Statement

This open-label study (NCT01669096) was conducted at the Centre for Vaccinology (CEVAC), Ghent University Hospital, Belgium, between August 2012 and May 2013. The protocol, its amendments, and other relevant study documentation were approved by the Ghent University Hospital’s Institutional Review Board, and the study was conducted in accordance with International Conference on Harmonisation Guideline for Good Clinical Practice, the Declaration of Helsinki, and all applicable regulatory requirements.

## Author Contributions

RB, LM, GL-R, VB, EJ, TE, PG, and RM were involved in the design of the study. RB, LM, GL-R, VB, FC, TE, PG, and RM participated in data collection. RB, LM, GL-R, and FC performed the analyses. RB, LM, GL-R, VB, FC, MC, EJ, TE, PG, and RM were involved in data interpretation. All authors were involved in drafting the manuscript or revising it for important intellectual content, have approved it, and have agreed to be accountable for all aspects of the work presented in it.

## Conflict of Interest Statement

All authors completed the ICMJE Form for disclosure of potential conflicts of interest and declared that the following interests are relevant to the submitted work. EJ, LM, MC, PG, RB, RM, and VB are employees of GSK group of companies. EJ, PG, RB, and RM report ownership of shares and/or restricted shares of the GSK group of companies. GL-R and FC report that the institutes in which they are based, Ghent University and Ghent University Hospital, have received grants from the GSK group of companies for the conduct of the study and they have received payments from the GSK group of companies for consulting in the field of vaccine adjuvants and unrelated to the current study. TE reports no conflicts of interest.

## Footnotes

^1^https://www.ncbi.nlm.nih.gov/geo/.

^2^https://cran.r-project.org/src/contrib/Archive/tmod/.

## Abbreviations

AE, adverse event; BCG, Bacillus Calmette–Guérin; BTM, blood transcription module; GS^+^/GS^−^, gene signature positive/negative; LLOQ, lower limit of quantification; Mtb, *Mycobacterium tuberculosis*; PBMC, peripheral blood mononuclear cell; pIMD, potential immune-mediated disease; SAE, serious adverse events; TB, tuberculosis; Th1, T-helper 1.
