# Supplementary figures and images for: Adjuvant-Associated Peripheral Blood mRNA Profiles and Kinetics Induced by the Adjuvanted Recombinant Protein Candidate Tuberculosis Vaccine M72/AS01 in Bacillus Calmette–Guérin-Vaccinated Adults
Source: Front Immunol. 2018 Mar 26;9:564. doi: 10.3389/fimmu.2018.00564 (PMC5879450; doi:10.3389/fimmu.2018.00564)

SUPPLEMENTAL MATERIAL

Figure S1

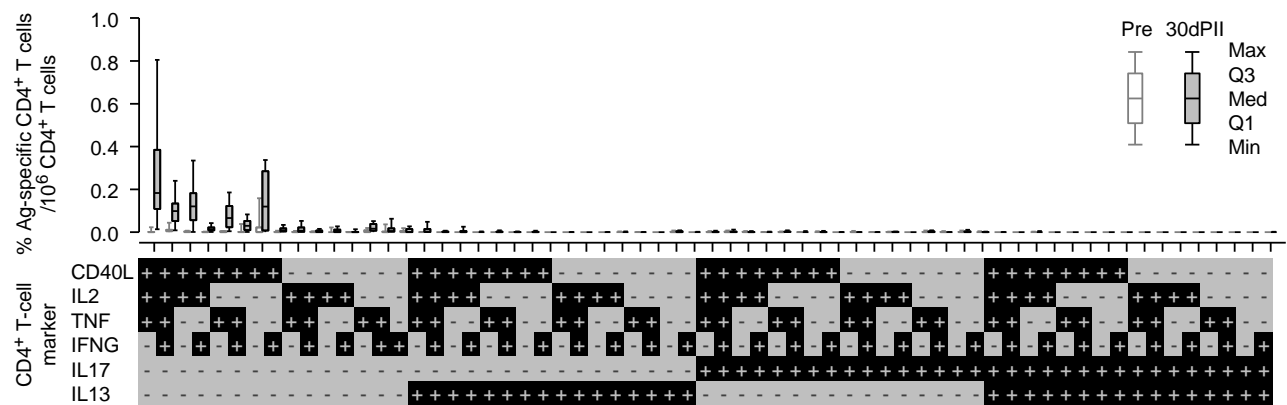

Supplement: Supplementary file 1 [file image_1.PDF]
