# Supplementary material for: Adjuvant-Associated Peripheral Blood mRNA Profiles and Kinetics Induced by the Adjuvanted Recombinant Protein Candidate Tuberculosis Vaccine M72/AS01 in Bacillus Calmette–Guérin-Vaccinated Adults
Source: Front Immunol. 2018 Mar 26;9:564. doi: 10.3389/fimmu.2018.00564 (PMC5879450; doi:10.3389/fimmu.2018.00564)
Supplement: Supplementary file 2 [file table_1.docx]

# Supplemental Material

**Peripheral-blood mRNA profiles and kinetics induced by the adjuvanted recombinant-protein candidate tuberculosis vaccine M72/AS01 in BCG-vaccinated adults.**

Robert A. van den Berg, Laurane De Mot, Geert Leroux-Roels, Viviane Bechtold, Frédéric Clement, Margherita Coccia, Erik Jongert, Thomas G. Evans, Paul Gillard and Robbert G. van der Most.

## Table S1

List of probe sets and corresponding gene targets in Cluster A and Cluster B.

| Cluster A | | | | | | | | | |
| --- | --- | --- | --- | --- | --- | --- | --- | --- | --- |
| Probe set ID | Gene | | Probe set ID | | Gene | | Probe set ID | | Gene |
| 222700_at | *ATL2* | | 241834_at | | *IPW* | | 229751_s_at | | *PUS7L* |
| 1559097_at | *C14ORF64* | | 244881_at | | *LMLN* | | 225189_s_at | | *RAPH1* |
| 227301_at | *CCT6P1* | | 228062_at | | *NAP1L5* | | 212482_at | | *RMND5A* |
| 227313_at | *CNPY4* | | 222290_at | | *OR2A9P* | | 223548_at | | *SWT1* |
| 205684_s_at | *DENND4C* | | 204853_at | | *ORC2L* | | 227205_at | | *TAF1* |
| 227696_at | *EXOSC6* | | 212593_s_at | | *PDCD4* | | 1568594_s_at | | *TRIM52* |
| 212991_at | *FBXO9* | | 205380_at | | *PDZK1* | | 231479_at | | *TTC33* |
| 224481_s_at | *HECTD1* | | 242323_at | | *PLA2G12A* | | 225089_at | | *USP40* |
| 217328_at | *IL23A* |  | |  | |  | |  | |
| Cluster B | | | | | | | | | |
| Probe set ID | Gene | | Probe set ID | | Gene | | Probe set ID | | Gene |
| 202387_at | *BAG1* | | 202471_s_at | | *IDH3G* | | 219202_at | | *RHBDF2* |
| 224727_at | *C19ORF63* | | 208436_s_at | | *IRF7* | | 226453_at | | *RNASEH2C* |
| 213571_s_at | *EIF4E2* | | 1560396_at | | *KLHL6* | | 231635_x_at | | *RNF31* |
| 212370_x_at | *FAM21A/B/C* | | 217165_x_at | | *MT1F* | | 205241_at | | *SCO2* |
| 214946_x_at | *FAM21A/B/C/D* | | 212185_x_at | | *MT2A* | | 223980_s_at | | *SP110* |
| 218023_s_at | *FAM53C* | | 209124_at | | *MYD88* | | 209762_x_at | | *SP110* |
| 207574_s_at | *GADD45B* | | 226707_at | | *NAPRT1* | | 200977_s_at | | *TAX1BP1* |
| 202680_at | *GTF2E2* | | 40640_at | | *NCAPH2* | | 48531_at | | *TNIP2* |
| 215313_x_at | *HLA-A* | | 214084_x_at | | *NCF1C* | | 202702_at | | *TRIM26* |
| 213932_x_at | *HLA-A* | | 219084_at | | *NSD1* | | 38964_r_at | | *WAS* |
| 209140_x_at | *HLA-B* | | 211012_s_at | | *PML* | | 204022_at | | *WWP2* |
| 208812_x_at | *HLA-C* | | 1553587_a_at | | *POLE4* | | 201531_at | | *ZFP36* |
| 203932_at | *HLA-DMB* | | 206687_s_at | | *PTPN6* | |  | |  |
| 200598_s_at | *HSP90B1* | | 227635_at | | *RBBP6* | |  | |  |
